# Analysis of the Clinical Value of Laparoscopic Sacrocolpopexy to Support the Posterior Compartment in Women with Multicompartment Prolapse Including Rectocele

**DOI:** 10.3390/jcm13175051

**Published:** 2024-08-26

**Authors:** Simone Aichner, Andreas Studer, Janine Frey, Christine Brambs, Jörg Krebs, Corina Christmann-Schmid

**Affiliations:** 1 Department of Urogynecology, Women’s Hospital, Cantonal Hospital of Lucerne, Spitalstrasse, 6000 Lucerne, Switzerland; 2 Swiss Paraplegic Research, Guido A. Zäch Strasse 4, 6207 Nottwil, Switzerland

**Keywords:** pelvic organ prolapse, bowel symptoms, laparoscopy, prolapse symptoms, pelvic floor surgery

## Abstract

**Background/Objectives**: Laparoscopic sacrocolpopexy is regarded as the gold standard treatment for apical or multicompartment prolapse, predominantly with anterior compartment descent. However, the optimal surgical approach for concurrent rectocele is still debated. The aim of this study was to evaluate the effectiveness of nerve-sparing laparoscopic sacrocolpopexy in managing multicompartment prolapse with concurrent rectocele (≥stage II), analyzing the anatomical outcomes, the necessity for concomitant or subsequent posterior repair, and the impact on bowel function in women undergoing surgery. **Methods**: Data from all women who underwent laparoscopic sacrocolpopexy with or without posterior repair between 01/2017 and 07/2022 for symptomatic multicompartment prolapse, including apical and posterior compartment descent ≥ stage II, were retrospectively evaluated. All women underwent a standardized urogynecological examination, including assessment of genital prolapse using the POP-Q quantification system, and completed the German-validated Australian Pelvic Floor Questionnaire before and after surgery (6–12 weeks). Preoperative anatomic support and bowel symptoms were compared with postoperative values. **Results**: In total, 112 women met the criteria for surgical correction. The majority (87%) had stage II posterior descent, with only 10% undergoing concurrent posterior repair during laparoscopic sacrocolpopexy. Significant (*p* < 0.001) objective improvement was seen for all compartments post- compared with preoperatively (Ba: 0 (−1/2) vs. −3 (−3/−2), C: −1 (−2/0) vs. −8 (−12/−7), Bp: 0 (−1/0) vs. −3 (−2/−2); (median (25%/75% quartiles)). Subsequent surgery for persistent rectocele and/or stool outlet symptoms was required in 4% of cases. Most bowel-specific questions in the German-validated Australian Pelvic Floor Questionnaire showed significant improvement (*p* < 0.001). **Conclusions**: Nerve-sparing sacrocolpopexy alone appears to be a suitable surgical approach to correct multicompartment prolapse, including a rectocele ≥ stage II, and results in a reduction of objective signs and symptoms of pelvic organ prolapse.

## 1. Introduction

Pelvic organ prolapse (POP) is a common condition of increasing importance due to demographic development, as it predominantly affects older women. During urogynecological examination, some degree of POP is found in up to 50% of all women, while 3–6% report bothersome symptoms and opt for medical treatment. Pelvic organ prolapse symptoms can affect women of every age, but the peak incidence occurs in women aged 70 to 79 years [1,2]. Anterior compartment prolapse occurs most frequently and is found twice as often as posterior compartment prolapse and three times more often than apical compartment descent (of the uterus/cervix or the vaginal vault post-hysterectomy) [3,4]. As POP is a dynamic condition, a strict distinction is often not possible, and thus approximately two out of three women affected by POP show a multicompartment prolapse [5]. POP is an anatomical disorder causing bothersome symptoms and functional impairment of the pelvic organs. Women with these disorders suffer physical and emotional distress, leading to a significant decrease in health-related quality of life, and surgical management can be indicated [6]. Among the available surgical approaches, laparoscopic sacrocolpopexy (SCP) is regarded as the gold standard treatment for women with apical or multicompartment prolapse [7,8]. Compared with vaginal interventions, as an alternative procedure, it is associated with a lower risk of prolapse awareness and a reduced rate of recurrent prolapse requiring repeat surgery. The laparoscopic approach is preferable due to its shorter operating time compared with the robotic approach and its shorter admission compared with the open approach [9]. In the case of an isolated rectocele, various surgical techniques are available, including transvaginal, transanal, transperineal, or transabdominal approaches, depending on the type of rectocele and its involvement of the mid or low rectum [10]. However, in the case of a multicompartment prolapse with a concurrent rectocele, the optimal surgical management remains debatable. To date, there are conflicting discussions in the literature regarding whether a concurrent rectocele can be treated with an SCP alone or whether a concomitant posterior repair (PR) should be performed. Furthermore, it must be acknowledged that achieving an adequate anatomical correction of posterior compartment prolapse does not always lead to a functional improvement of bowel symptoms [11].

There are data demonstrating long-term objective improvement in posterior support with resolution of obstructive bowel symptoms after SCP regardless of whether PR was performed concurrently [12,13,14,15]. Wagner et al. stated in their prospective analysis that SCP is associated with excellent long-term anatomical correction of the anterior and apical compartments, with the highest recurrence rates of 19% in the posterior compartment [16]. A recently published retrospective study suggests that concurrent PR should be performed at the time of SCP, as this surgical approach leads to significantly better resolution of subjective bulge symptoms and lower reoperation rates for symptomatic rectocele compared with SCP alone [17]. To date, there is no consensus on the optimal surgical strategy for combined apical and posterior vaginal prolapse.

Therefore, the primary aim of this study was to evaluate whether nerve-sparing laparoscopic sacrocolpopexy (NS-SCP) alone would achieve successful support of the posterior compartment in women with multicompartment prolapse including a rectocele of at least stage II. Secondarily, the study aimed to demonstrate the improvement of pelvic floor function, especially of bowel function, related to the anatomical correction of posterior vaginal descent.

## 2. Materials and Methods

### 2.1. Study Design

In this retrospective exploratory cohort study, we evaluated the medical records of all women who underwent NS-SCP with or without (sub)total hysterectomy for apical or multicompartment prolapse with a concurrent rectocele of at least stage II (International Continence Society (ICS) POP-Q quantification system [18]) from January 2017 to July 2022 at the Department of Urogynecology of our tertiary referral hospital. This study had been approved by the local ethics committee (Project-ID 2022-01346). All included women had signed a general study consent.

Surgery was performed for symptomatic apical or multicompartment prolapse. Women received either NS-SCP or NS-SCP plus PR at each surgeon’s discretion, depending on preoperatively reported bothersome stool outlet obstruction with digitation and/or a deep rectal pocket (descent 1–3 cm beyond the hymenal ring).

During the preoperative visit, a complete gynecological and obstetric history was recorded. All women underwent a standardized gynecological and urogynecological examination, including the assessment of the prolapse stage preoperatively and postoperatively (6–12 weeks). To determine subjective symptoms, all women completed the German-validated Australian Pelvic Floor Questionnaire (APFQ), which contains domains of bladder, bowel, prolapse, and sexual function pre- and postoperatively (6–12 weeks). The bowel function domain contains questions regarding constipation, laxative use, stool outlet obstruction, including digital evacuation, fecal incontinence, and experienced bother regarding bowel symptoms. The higher the rated scores, the more bothersome the symptoms are [19,20]. Postoperative objective success was defined as follows: POP-Q point C < −4 and <−1 for point Ba and Bp, respectively. In the event of persistent bothersome posterior descent during postoperative evaluation, a PR was performed as a two-step approach and the corresponding data were recorded.

### 2.2. Surgical Technique

All women included in this study underwent a standardized NS-SCP with or without a concomitant PR, as previously described and published [21]. For NS-SCP, the peritoneum was carefully opened at the sacral promontory, and the longitudinal ligament was dissected while avoiding damage to the middle sacral veins, which marked the end of the medial dissection. Throughout this process, the inferior hypogastric nerve (IHN) medial to the ligament, which is crucial for maintaining bladder, bowel, and sexual function, was continuously visualized and preserved. The peritoneum was then superficially opened from the sacral promontory to the vaginal apex along the right pelvic sidewall medial to the ureter. Deep dissection was avoided to preserve the IHN, especially at the remnant uterosacral ligament. The vesicovaginal space was opened and dissected to the bladder neck, with lateral dissection limited by the vesicouterine ligament vessels. The rectovaginal space was opened and carefully dissected to the cranial edge of the perineal body at the level of the levator ani muscle. The Y-shaped mesh (EndoGYNious polypropylene mesh produced by A.M.I.^®^, Feldkirch, Austria) was anteriorly and posteriorly fixed to the vaginal wall with several stitches (self-absorbing thread, PDS 2-0) without suturing the levator ani muscle posteriorly. The most distal knot was placed approximately 1.5 cm above the most distal edge of the mesh. The peritoneal tunnel was joined with dissection of the superficial layer of the right uterosacral ligament, preserving its deep nervous portion as described by Ercoli et al. [22]. The polypropylene mesh was fixed at the sacral promontory, ensuring the IHN was medial to the fixation points.

In the case of concomitant PR, which was performed after NS-SCP, a transvaginal posterior colporrhaphy was performed in the most distal vaginal portion. For this purpose, we made a vertical incision in the vaginal wall and dissected the vaginal mucosa, preserving the rectovaginal fascia, starting from the hymenal fringe, and extending 3–4 cm apically (with palpable overlap of approximately 1 cm with the mesh). Special care was taken to avoid cutting the distal knot of the SCP during the PR procedure. To reinforce the posterior wall, a midline fascial plication of the rectovaginal septum and the vaginal muscularis was performed using absorbable sutures (PDS 2-0). The excess vaginal epithelium was removed (as little as possible to avoid vaginal stenosis), and the edges of the vaginal tissue were reassembled [23]. Surgery was conducted by the senior author or one of her urogynecological fellows under her supervision.

### 2.3. Statistical Analysis

Data were calculated as mean and standard deviation (SD) or median with lower (LQ) and upper quartiles (UQ) or frequency and percentage.

Depending on the type of data, the differences between pre- and postoperative values were investigated using the Wilcoxon-signed ranks test, the McNemar test, or the related-samples marginal homogeneity test with Bonferroni correction for multiple testing. The cohort was divided into two groups depending on the severity of bother regarding bowel symptoms before surgery. Group A included all women with no or only mild complaints (answered with “not at all” or “slightly” to the question regarding experienced bother, question 27 of the APFQ), while Group B included all women with moderate or severe symptoms (answered with “moderately” or “greatly”). Preoperative differences between the groups were investigated using the unpaired Student’s *t*-test, the Mann–Whitney U test, or the chi-square test. The changes over time were investigated using the Brunner non-parametric (rank-based) analysis of variance of longitudinal data [24]. Post-hoc testing was performed using the Wilcoxon signed-ranks test with Bonferroni correction.

Statistical analyses were performed using the SPSS software (Version 25, IBM, Somers, NY, USA) or the R software environment (Version 4.3.2, Copyright 2023, The R Foundation for Statistical Computing, Vienna, Austria). A *p*-value of ≤0.05 was considered significant.

## 3. Results

During the study period of 5.5 years, 112 women were included, and their data were evaluated. Of all the women included a total of six patients did not appear for the postoperative visit, while three of them returned the completed APFQ. Of the 106 postoperatively seen patients, subjective data for six women are missing, as they did not complete the APFQ.

Table 1 summarizes the baseline demographic and clinical parameters as well as previous pelvic floor surgeries and the type of performed surgery. Included women were either affected by posterior POP stage II (97/112, 87%) or stage III (15/112, 13%). Seventeen of 112 women (15%) had experienced POP recurrence before the baseline visit. One woman out of 17 was affected by recurrent posterior vaginal descent after previous posterior colporrhaphy. Three other women had anterior and posterior recurrent vaginal descent after anterior and posterior colporrhaphy (2/3) or rectopexy (1/3). The other women had recurrence most frequently of the anterior and/or apical compartment after anterior colporrhaphy and/or sacrospinous fixation or laparoscopic sacrocolpopexy or hysteropexy. All women underwent laparoscopic NS-SCP with or without a (sub-)total hysterectomy or sacrohysteropexy. A minority of women (11/112, 10%) had a concurrent PR at the time of surgery. Preoperative bothersome stool outlet symptoms with/without digitation were reported in six cases, a deep palpable rectal pocket in three cases, and a combination of these in two of these patient groups.

### 3.1. Objective Outcome Measurements

A significant (*p* < 0.001) objective improvement of POP values regarding all three compartments (Ba, C, and Bp) was seen postoperatively compared with preoperatively, as shown in Table 2.

Point C was <−4 in all women postoperatively. However, in nine women, the POP-Q value for point Ba was −1 or 0; in three women, the POP-Q value for point Bp was −1 or 0; and in four women, Ba and Bp were −1 or 0. In no case were Ba or Bp > 0. In the group of women with PR at the time of surgery, the POP-Q value for Bp was −3 in eight women and −2 in two women (data missing from one woman). In five out of 96 women (5%), PR was indicated due to persistent rectocele and bothersome stool outlet symptoms and performed in a subsequent surgery in four women (in one woman, PR was planned but cancelled because of remission of symptoms).

### 3.2. Subjective Outcome Measurements

Table 3 summarizes the bowel function data pre- compared with postoperatively. A significant (*p* < 0.05) improvement was seen in most preoperatively reported symptoms, such as straining at defecation, fecal urgency, involuntary loss of normal stool, the sensation of incomplete evacuation, and the need for self-digitation to help empty the bowel. A significant (*p* = 0.02) difference was also observed regarding laxative use, which was more frequent postoperatively. No changes in symptoms such as constipation and involuntary loss of wind and fluid stools were observed. Overall, the experienced bother for bowel symptoms was significantly (*p* < 0.001) lower postoperatively.

In line with the objective improvement of POP-values, subjective reported POP-symptoms improved significantly (*p* < 0.001) postoperatively as shown in Table 4.

Regarding sexual function, no improvement (*p* = 0.9) was observed in the percentage of sexual problems or dyspareunia from preoperative to postoperative. Concerning bladder function, a significant (*p* < 0.001) improvement in overactive bladder (OAB) symptoms was observed together with an improvement in bladder voiding (*p* < 0.001), while no difference (*p* = 0.9) was observed either regarding stress urinary incontinence symptoms or incontinence episodes during stress tests.

### 3.3. Subgroup Analysis

Demographic data such as age, birth weight, parity, and the proportion of women with adiposity did not differ between Group A with no or mild preoperatively reported bowel symptoms and Group B with moderate or distinct symptoms (*p* > 0.05 for all items). Table 5 demonstrates that there were no differences regarding the preoperative POP-Q values Bp, gh, and pb between the two groups.

Table 6 summarizes the answers given to the questions regarding bowel symptoms in the APFQ. Group B had more preoperative bowel complaints, and an improvement of symptoms was generally only seen in Group B, except for laxative use (mild worsening), constipation, and involuntary loss of normal stool (showing no change). A significant improvement (*p* < 0.001) was observed in group B regarding straining to empty and stool urgency pre- to postoperatively. Furthermore, there was a decrease in vaginal/perineal digitation to assist defecation as well as bother from bowel symptoms (*p* < 0.001). Group A showed nearly no difference between preoperative and postoperative values, except for a mild improvement in symptoms of fecal urgency and mild worsening regarding constipation, wind incontinence, and the sensation of incomplete bowel emptying.

## 4. Discussion

This retrospective study clearly demonstrates that performing NS-SCP to correct a multicompartment prolapse including posterior vaginal descent of at least stage II leads to an excellent anatomical correction of the apical, anterior, as well as posterior compartment. These findings are in line with existing data in the literature, showing similar results [13,25]. We found a satisfactory surgical success of the apical compartment (100%), while mild objective signs of prolapse were observed anteriorly and/or posteriorly in a few women. Even though a distinct objective descent was present, Ba and/or Bp was ≤0 in all women, which can be considered surgical success, as bothersome symptoms and the impact on pelvic organ function and quality of life most commonly first occur with a descent beyond the hymenal ring [26]. Together with anatomical correction, subjectively reported symptoms improved significantly pre- to postoperatively.

Interestingly, only one-half of all investigated women affected by rectocele of at least stage II reported bothersome bowel symptoms preoperatively. This suggests that a rectocele is not necessarily associated with bowel symptoms, which could be previously shown [27]. The lack of difference in Bp values between the two groups with and without bowel symptoms preoperatively in the subgroup analysis supports this thesis.

Although posterior descent seems not to lead to bowel symptoms in all cases, our findings suggest that, if present, they improve with surgical correction. Fortunately, on the other hand, surgical intervention did not lead to de novo bowel symptoms in cases of no preoperatively observed complaints in our cohort. This may, among other factors, be attributed to the performed surgical technique, in which the mesh is sutured to the vaginal wall and not to the levator ani muscle, which has been discussed previously [28]. The latter surgical technique is controversial, because fixation of the muscle may cause increased pain and lead to bowel symptoms [29], and experts tend to prefer a fixation of the mesh to the posterior vaginal surface alone [30]. Moreover, the implemented nerve-sparing approach is recognized for its favorable outcomes in preventing de novo pelvic floor dysfunction, including bowel complaints [21].

We found a significant improvement in symptoms such as straining to defecate, fecal urgency, incontinence for normal stool, feeling of incomplete evacuation, and need for digital assistance to defecate. No difference was found in constipation symptoms, and more laxative use was found postoperatively, which is evident as this was one of our postoperative recommendations, based on the known fact that SCP is generally associated with constipation in the early postoperative phase [29]. No difference was found for the involuntary loss of wind and fluid stool. This is an anticipated fact, as it is known that severe anal incontinence is not associated with posterior compartment prolapse [31].

The results of our retrospective study indicate that NS-SCP alone leads to adequate correction of the posterior compartment and results in resolution of bowel complaints, if any were present. The circumstance that only a minority of all women of this cohort had primary (10%) or subsequent (4%) PR suggests that simultaneously performed PR together with an SCP in women with concomitant rectocele cannot be justified automatically.

Our results are consistent with previous findings, suggesting that concomitant PR does not lead to an additional benefit [32]. To support these statements, a comparison of objective and subjective outcomes between patients with SCP alone and with SCP plus PR would be interesting. The small sample size in the group of SCP plus PR in our cohort does not allow such comparisons. A limitation of our study is the vaguely defined criteria for concomitant PR during NS-SCP. Although the small overall proportion and low percentage of secondary PR procedures suggest that a persistent, symptomatic posterior defect following NS-SCP is not a major concern, it remains debatable to what extent concomitant PR influences postoperative results of NS-SCP.

The most limiting factor of our study is the short observation time due to the exploratory nature of this study. Based on the generally low recurrence rate of SCP [25], we extrapolate the ongoing favorable improvement in subjective symptoms without discernible changes in anatomical correction over time, which should be evaluated in further studies. The strength of the study is the relatively high sample size and the standardized surgical technique and clinical evaluation pre- and postoperatively. The use of the POP-Q quantification system and the standardized APFQ made it possible to generate highly reproducible, objective, and comparable results.

## 5. Conclusions

Based on the results of this study, from a cohort of 112 women, we can hypothesize that NS-SCP in women with symptomatic multicompartment prolapse, including rectocele, results in excellent anatomical correction and improvement in bowel symptoms, if present preoperatively. Simultaneous or secondary PR is rarely necessary. Pending confirmation of these results in further studies, these findings currently suggest that sacrocolpopexy alone is a reasonable treatment option for the above-described prolapse cases. Future research in a prospective setting is needed to further investigate its long-term efficacy and impact on quality of life.

## Figures and Tables

**Table 1:** Demographics, previous organ prolapse surgery, posterior compartment POP stage, and type of performed surgery.

Demographics	*n* = 112	POP-Stage, Posterior Compartment	*n* = 112
Age (years), mean ± standard deviation	61 ± 10	stage II (*n*/%)	97/87%
Parity, median (lower quartile/upper quartile)	2 (2/3)	stage III (*n*/%)	15/13%
Birth weight (g), mean ± standard deviation	3702 ± 566	**Type of performed surgery**	
Obesity (BMI > 30 kg/m^2^) (*n*/%)	15/13%	Laparoscopic sacrocolpopexy (*n*/%)	34/30%
**Previous pelvic organ prolapse surgery**		Lap. supracervical hysterectomy plus SCP (*n*/%)	53/47%
Anterior colporrhaphy (*n*/%)	12/11%	Lap. assisted vaginal hysterectomy plus SCP (*n*/%)	16/14%
Posterior colporrhaphy (*n*/%)	3/3%	Laparoscopic total hysterectomy plus SCP (*n*/%)	3/3%
Rectopexy (*n*/%)	1/1%	Vaginal hysterectomy plus SCP (*n*/%)	3/3%
Sacrospinous fixation, Mc Call (*n*/%)	5/4%	Laparoscopic sacrohysteropexy (*n*/%)	3/3%
Sacrocolpopexy (*n*/%)	1/1%	Concomitant PR (*n*/%)	11/10%
Sacrohysteropexy (*n*/%)	1/1%		

BMI: body mass index; POP: pelvic organ prolapse; SCP: sacrocolpopexy; PR: posterior repair. Lap.: Laparoscopic.

**Table 2:** Pre- and postoperative POP-Q values.

POP-Q Values	Preoperative *	Postoperative *	*p*-Value
	*n* = 112	*n* = 106	
Aa	0 (−1/2)	−3 (−3/−2)	<0.001
Ba	1 (0/2)	−3 (−3/−2)	<0.001
C/D	−1 (−2/0)	−8 (−10/−7)	<0.001
Ap	−1 (−1/0)	−3 (−3/−2.75)	<0.001
Bp	0 (−1/0)	−3 (−3/−2)	<0.001
TVL	10 (8/10)	10 (9/11)	0.0002
gh	4 (3/4)	4 (3/4)	0.0015
pb	3 (2/3)	3 (3/3)	0.7

* Median, 25%/75% quartiles, TVL: total vaginal length, gh: genital hiatus, pb: perineal body.

**Table 3:** Given answers regarding pre- and postoperative bowel function data in the APFQ.

	Preoperative *				Postoperative °				*p*-Value
	Never	Occasionally	Frequently	Daily	Never	Occasionally	Frequently	Daily	
straining to empty	24/21%	57/51%	19/17%	12/11%	36/35%	53/51%	11/11%	3/3%	<0.001
laxative use	99/88%	8/7%	2/2%	3/3%	74/72%	17/17%	4/4%	8/8%	0.022
constipation	64/58%	37/33%	7/6%	3/3%	53/52%	43/42%	4/4%	3/3%	0.9
wind leak	37/33%	38/34%	26/23%	11/10%	43/42%	41/40%	4/4%	3/3%	0.077
urgency to empty	59/53%	36/32%	16/14%	1/1%	69/67%	29/28%	4/4%	1/1%	0.011
leak of watery stool	79/71%	27/24%	5/5%	0	84/82%	16/16%	3/3%	0	0.143
leak of normal stool	101/90%	9/8%	2/2%	0	96/93%	7/7%	0	0	0.022
incomplete emptying	48/43%	35/31%	17/15%	12/11%	61/59%	28/27%	12/12%	2/2%	<0.001
digitation	71/63%	24/21%	2/2%	15/13%	80/78%	16/16%	4/4%	3/3%	0.022
	not at all	slightly	moderately	greatly	not at all	slightly	moderately	greatly	
bother due to bowel symptoms	52/46%	30/27%	9/8%	21/19%	71/69%	19/18%	10/10%	3/3%	<0.001

* number of persons (*n*/112) and percentage with following answers. ° number of persons (*n*/103) and percentage with following answers

**Table 4:** Given answers regarding pre- and postoperative prolapse symptoms in the APFQ.

	Preoperative *				Postoperative °				*p*-Value
	Never	Occasionally	Frequently	Daily	Never	Occasionally	Frequently	Daily	
sensation of vaginal bulging	18/16%	9/8%	27/24%	58/52%	91/88%	9/9%	2/2%	1/1%	<0.001
vaginal pressure/dragging sensation	9/8%	12/11%	21/19%	69/62%	90/87%	10/10%	3/3%	0	0.37
	not at all	slightly	moderately	greatly	not at all	slightly	moderately	greatly	
bother due to prolapse symptoms	5/5%	18/16%	38/34%	50/45%	93/90%	7/7%	2/2%	1/1%	<0.001

* number of persons (*n*/112) and percentage with following answers. ° number of persons (*n*/103) and percentage with following answers

**Table 5:** Preoperative POP-Q values for Group A (no/mild bowel symptoms) and Group B (moderate/distinct bowel symptoms).

Preoperative POP-Q Values	Group A *	Group B *	*p*-Value
	*n* = 52	*n* = 60	
Bp	0 (−1/0)	0 (−1/1)	0.9
gh	4 (3/4.75)	4 (3/4)	0.9
pb	3 (2/3)	3 (2/3)	0.8

* Median, 25%/75% quartiles, gh: genital hiatus, pb: perineal body.

**Table 6:** Pre- and postoperative bowel symptoms from the APFQ for Group A (no/mild bowel symptoms preoperatively) and Group B (moderate/distinct bowel symptoms preoperatively).

			Preoperative	Postoperative	*p*-Value
straining to	Group A	*n* = 52	2 (1/2)	2 (1/2)	ns
empty the bowel *	Group B	*n* = 60	2.5 (2/3)	2 (1.75/2)	0.0005
laxative use *	Group A	*n* = 52	1 (1/1)	1 (1/1)	ns
	Group B	*n* = 60	1 (1/1)	1 (1/2)	ns
constipation *	Group A	*n* = 52	1 (1/1)	1 (1/2)	ns
	Group B	*n* = 60	2 (1/2)	2 (1/2)	ns
wind leak *	Group A	*n* = 52	1.5 (1/2)	2 (1/2)	ns
	Group B	*n* = 60	2 (2/3)	2 (1/3)	ns
urgency to	Group A	*n* = 52	1 (1/2)	1 (1/1)	ns
empty the bowel *	Group B	*n* = 60	2 (1/3)	1 (1/2)	0.0014
leak of watery stool *	Group A	*n* = 52	1 (1/1)	1 (1/1)	ns
	Group B	*n* = 60	1 (1/2)	1 (1/1.25)	ns
leak of normal stool *	Group A	*n* = 52	1 (1/1)	1 (1/1)	ns
	Group B	*n* = 60	2.5 (2/3)	2 (1.75/2)	ns
incomplete	Group A	*n* = 52	1 (1/1)	1 (1/1.5)	ns
Emptying *	Group B	*n* = 60	2 (2/3))	2 (1/2.25)	ns
perineal/	Group A	*n* = 52	1 (1/1)	1 (1/1)	ns
vaginal digitation *	Group B	*n* = 60	2 (1/3.75)	1 (1/2)	0.002
bother due to	Group A	*n* = 52	1 (1/1)	1 (1/1)	ns
to bowel symptoms °	Group B	*n* = 60	2.5 (2/4)	1 (1/2)	<0.001

* reported answers (1: never, 2: occasionally, 3: frequently, 4: daily); median 25%/75% quartiles. ° reported answers (1: not at all, 2: slightly, 3: moderately, 4: greatly), median 25%/75% quartiles. ns: not significant (*p* > 0.05) in non-parametric analysis of variance.

## Data Availability

The raw data are available upon request from the corresponding author.
